# Supplementary material for: Quadrant darkfield for label-free imaging of intracellular puncta
Source: J Biomed Opt. 2024 Nov 29;29(11):116501. doi: 10.1117/1.JBO.29.11.116501 (PMC11605245; doi:10.1117/1.JBO.29.11.116501)
Supplement: Supplementary file 1 [file JBO_029_116501_SD001.pdf]

Supplementary material for

Quadrant-darkfield for label-free imaging of intracellular puncta

Tarek E. Moustafa[a], Rachel L. Belote[b,c], Edward R. Polanco[a], Robert L. Judson-Torres[b,d,e], and Thomas A. Zangle[a,b]*

[a]University of Utah, Department of Chemical Engineering, Salt Lake City, Utah, United States

[b]University of Utah, The Huntsman Cancer Institute, Salt Lake City, Utah, United States

[c]The Ohio State University, Department of Molecular Genetics, Columbus, Ohio, United States

[d]University of Utah, Department of Dermatology, Salt Lake City, Utah, United States

[e]University of Utah, Department of Oncological Sciences, Salt Lake City, Utah, United States

*Corresponding author: tzangle@chemeng.utah.edu

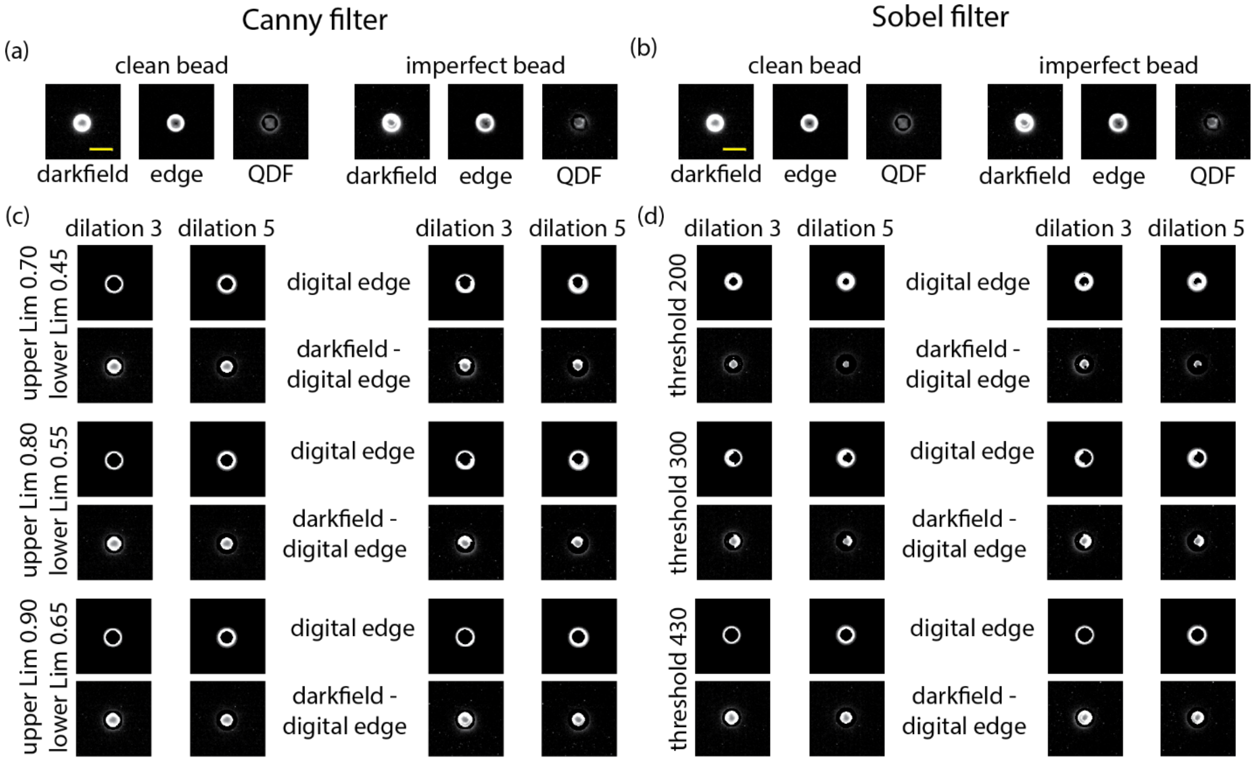

Figure S1. (a,b) Quadrant darkfield (QDF) compared to digital edge detection using (c) Canny filter and (d) Sobel filter. The effect of varying thresholds is displayed from top to bottom. The effect of varying dilation structure size is displayed left to right.

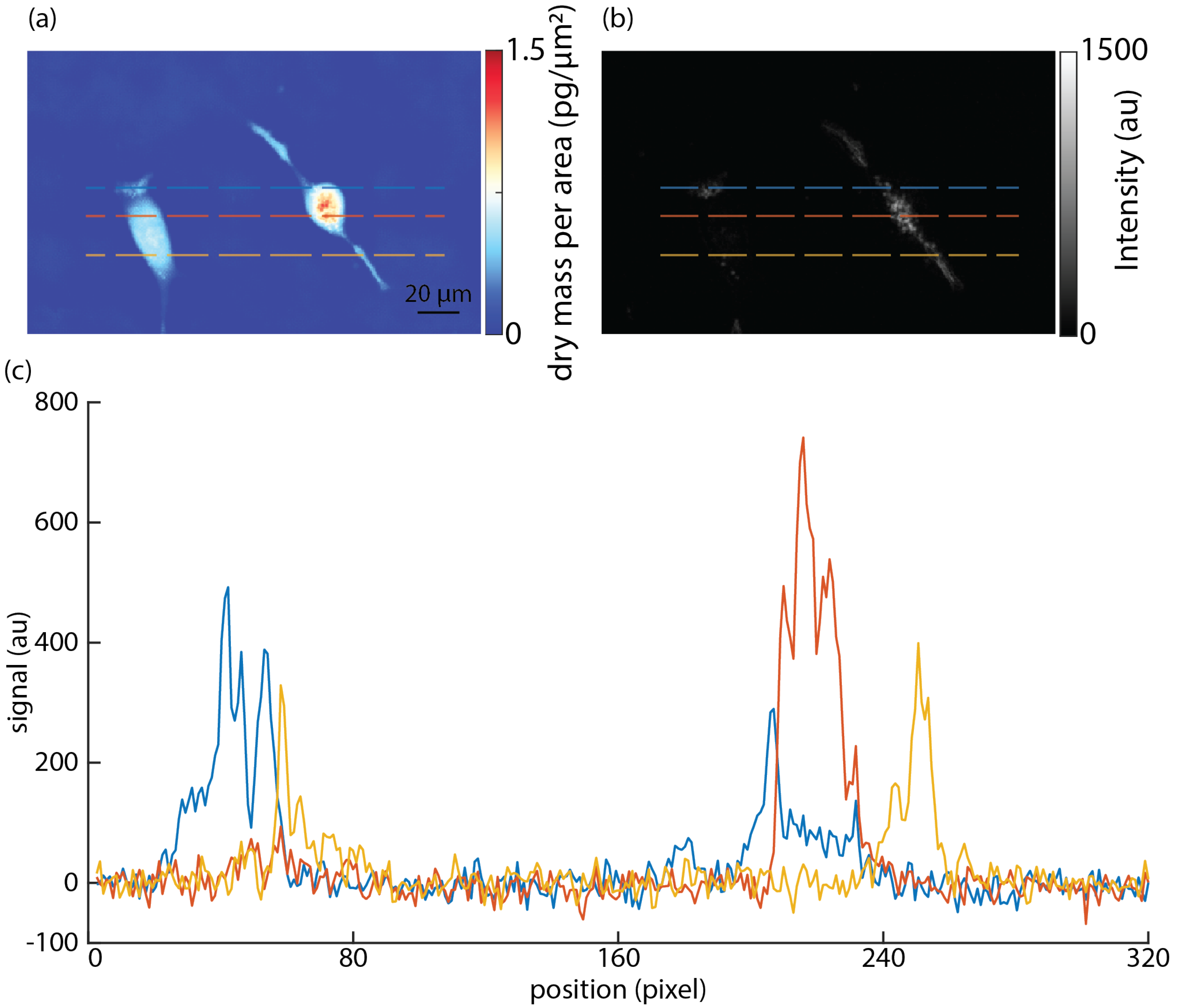

Figure S2. Zoomed view of MTG021 cells with (a) Quantitative phase imaging (QPI) and (b) QDF. (c) Cross section of QDF signal showcasing difference in signal between puncta and background as well as different puncta in each cell. Color of overlaid lines in (a) and (b) correspond to the color of plotted lines in (c) respectively.

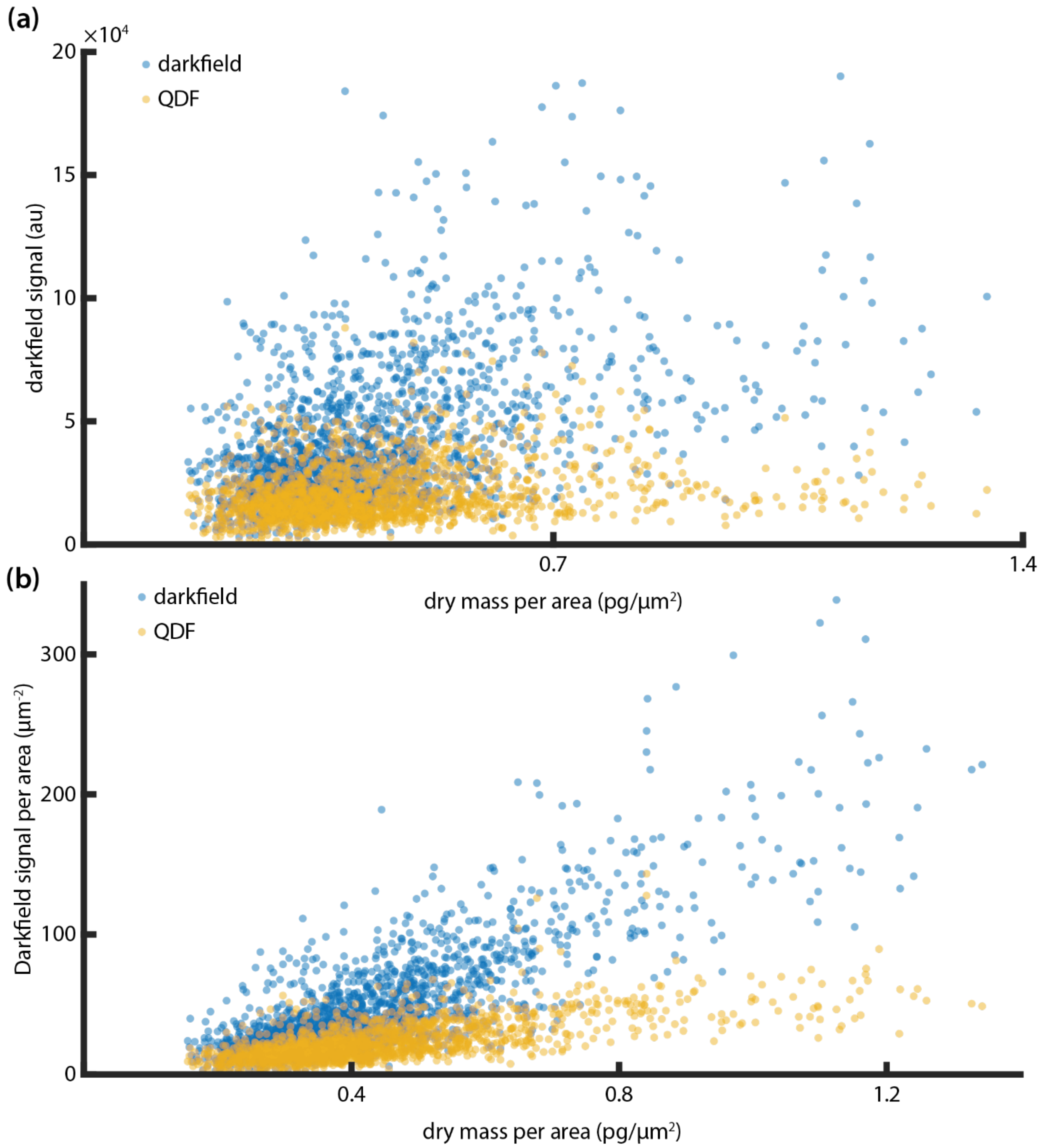

Figure S3. (a) Darkfield (blue) and QDF (yellow) signal versus dry mass per area as a proxy for shape change. (b) Darkfield (blue) and QDF (yellow) per area signal versus dry mass per area as a proxy for shape change.

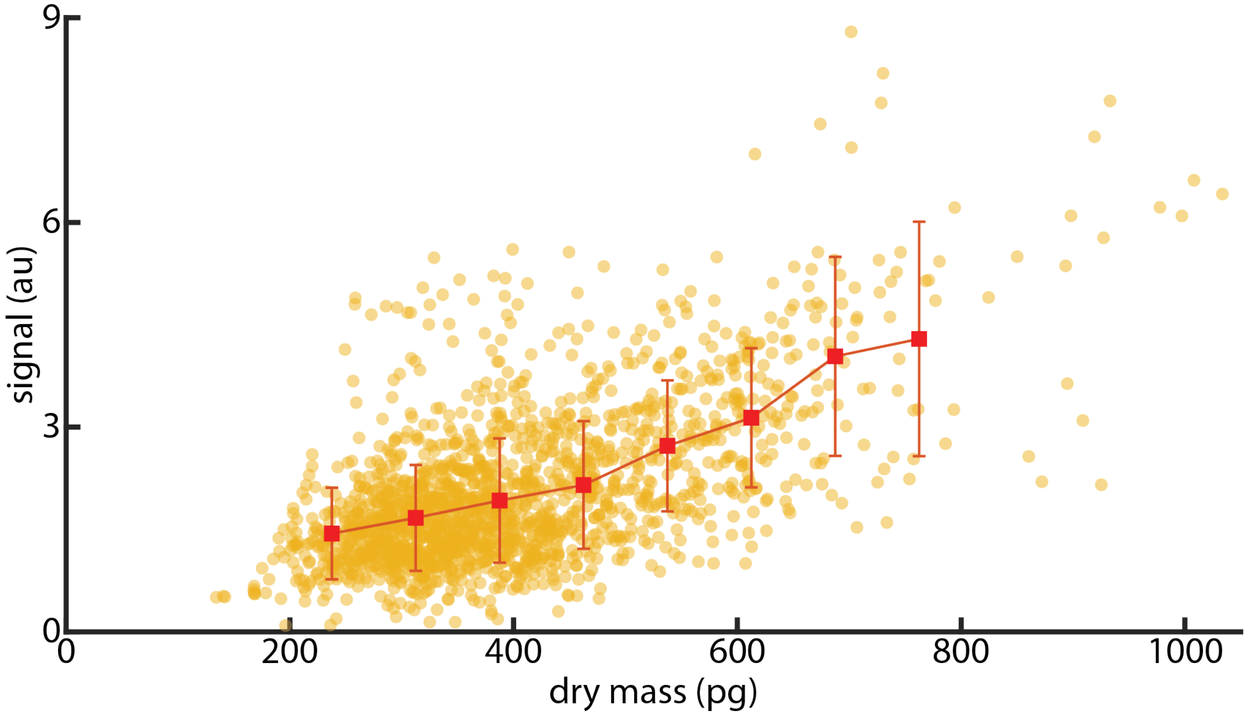

Figure S4. Scatter plot of QDF (yellow) signal against dry mass. Red points show mean of data in 75 pg wide bins. Error bars show the standard deviation of the binned data.

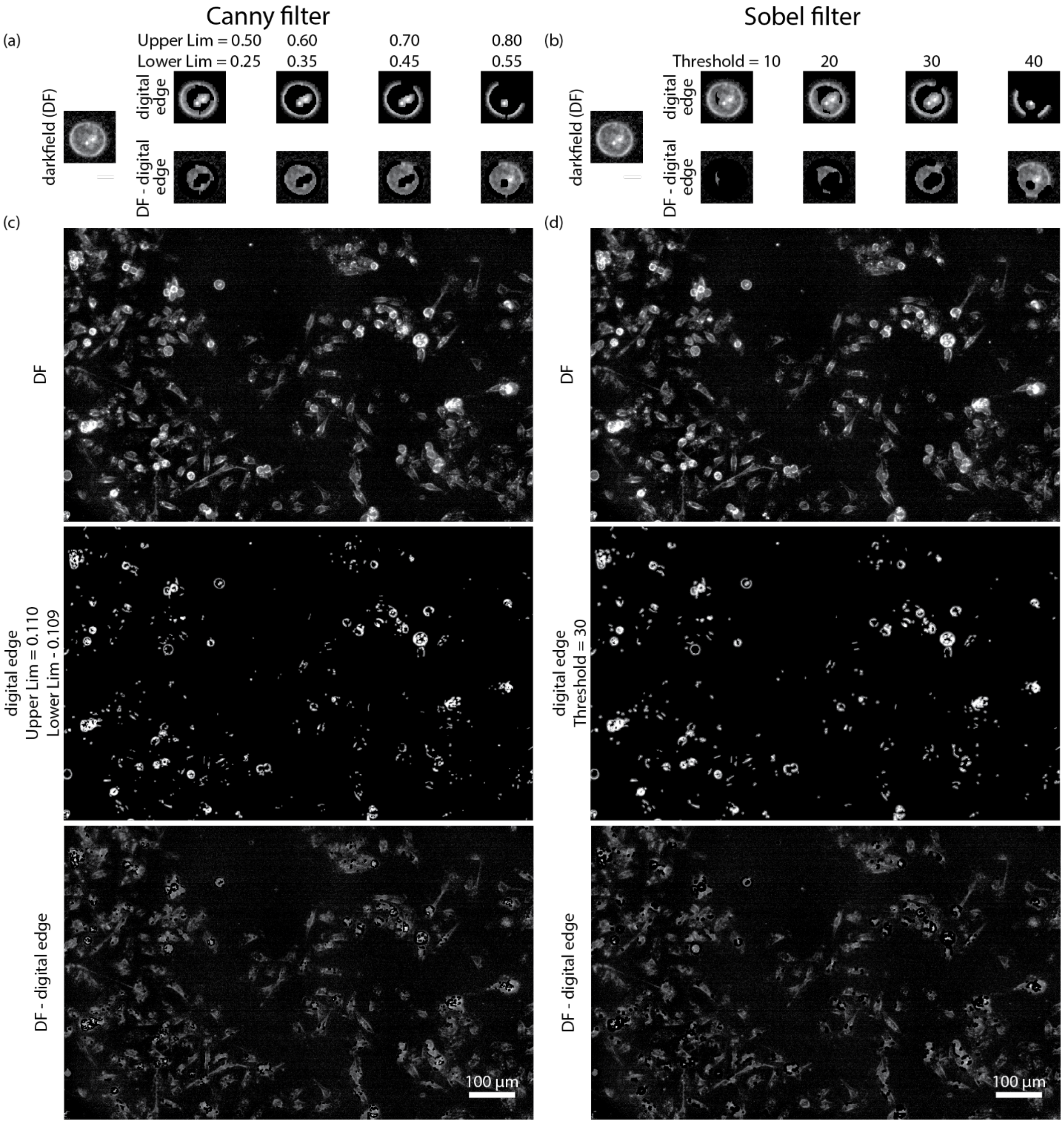

Figure S5. Zoomed field of view using (a) Canny filter and (b) Sobel filter. Full FoV using (c) Canny filter and (d) Sobel filter of MDA-MB-231 to separate darkfield signal into digital edge signal and darkfield signal minus digital edge.

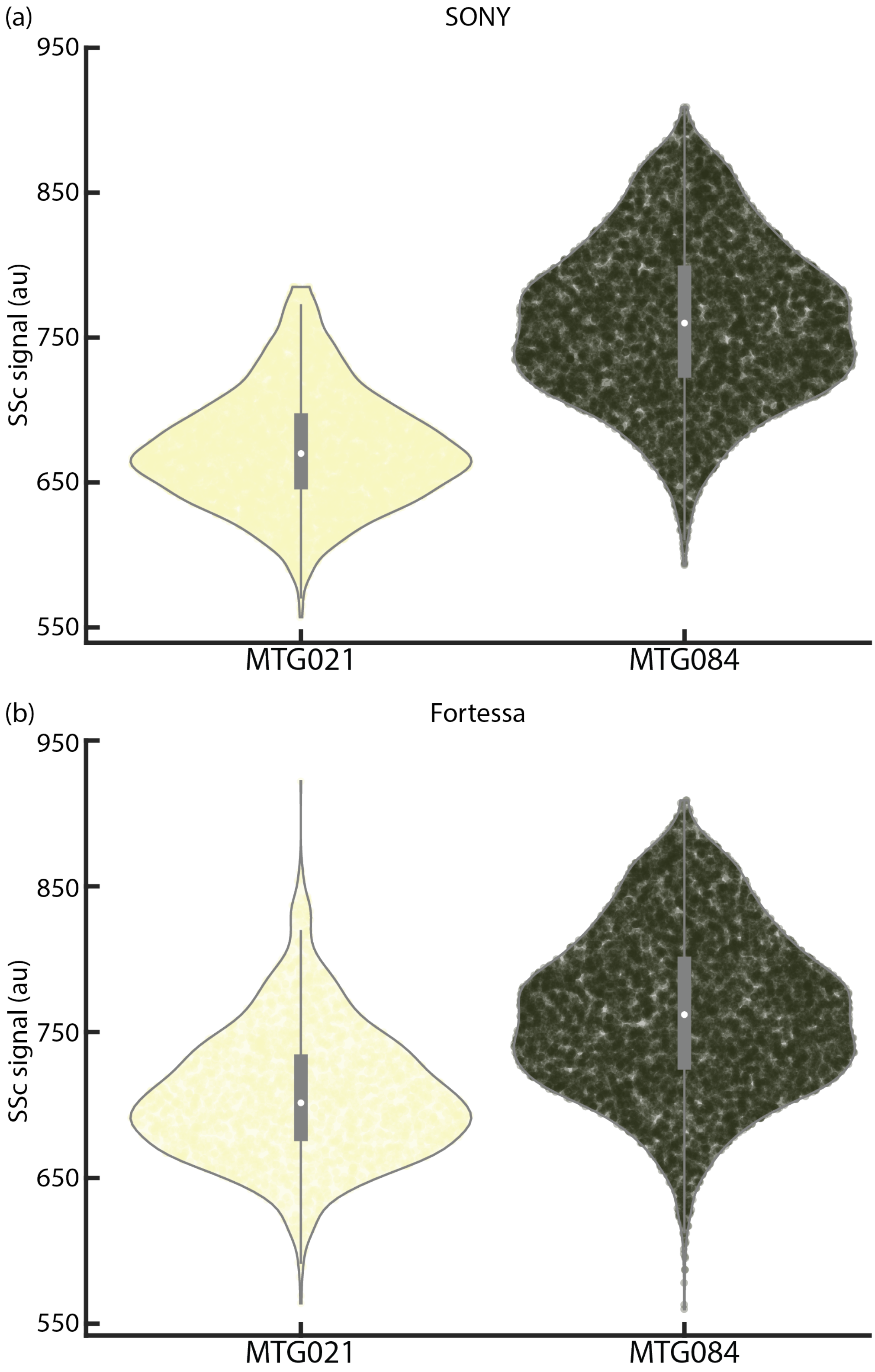

Figure S6. Distribution of side scatter signal for MTG021 and MTG084 from (a) SONY and (b) BD Fortessa flow cytometry devices.

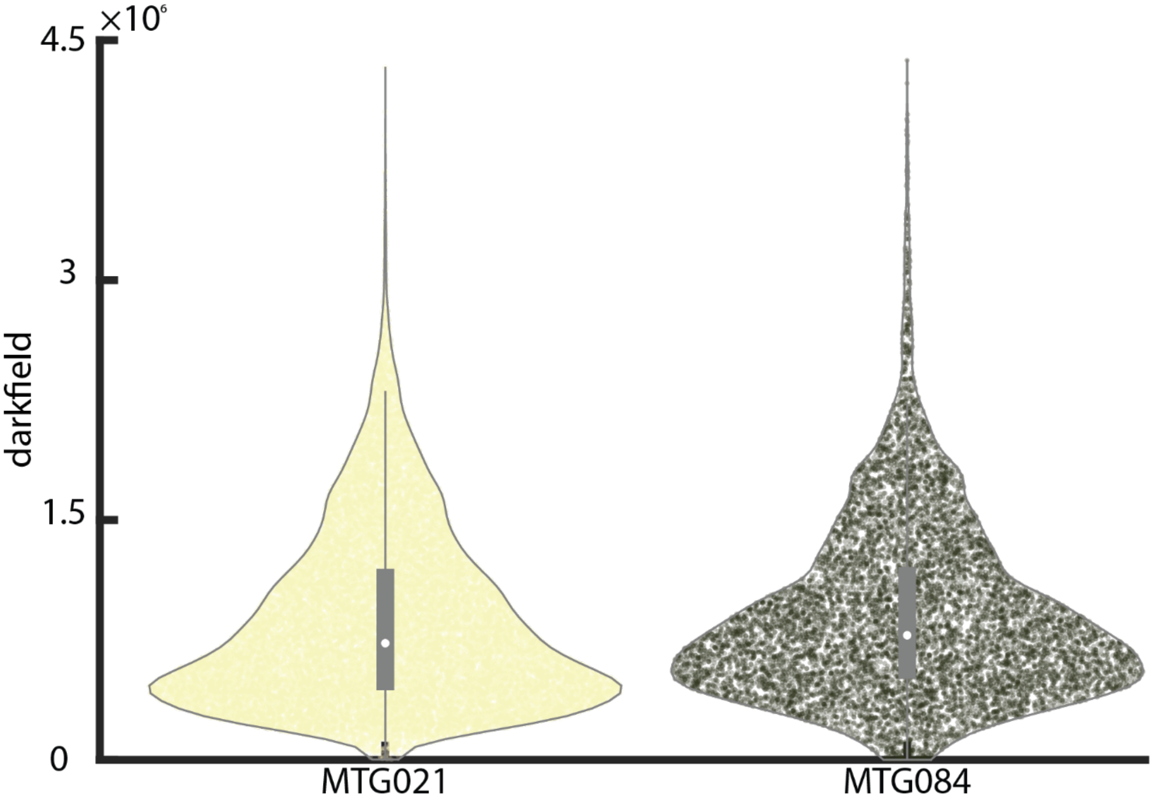

Figure S7. Distribution of darkfield signal from MTG021 and MTG084 cell lines.

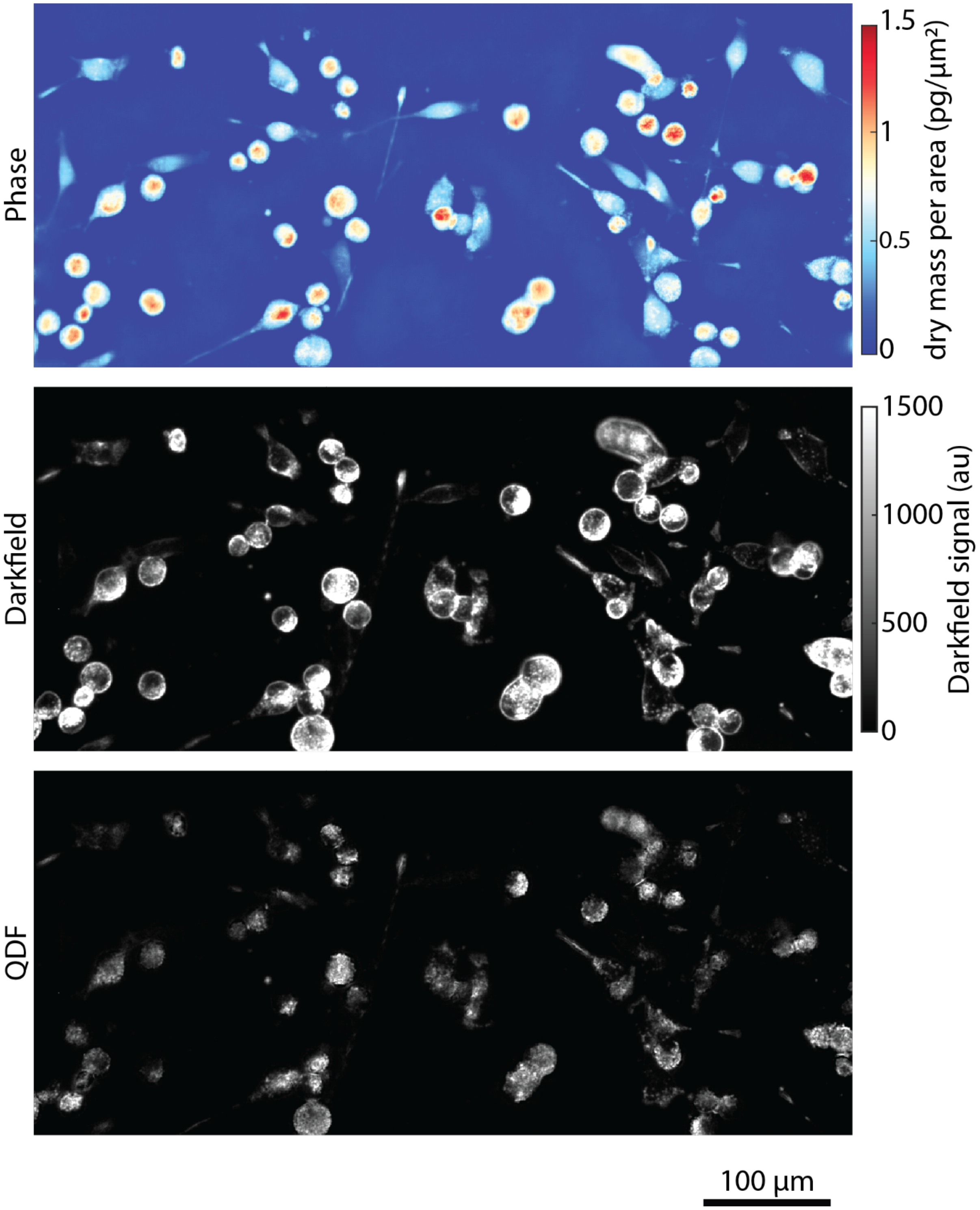

Figure S8. Phase (QPI), Darkfield and QDF images of MTG021 showing clearer localization of puncta in QDF.

Ideal QDF model and the need for a scaling factor

Here, we derive the scaling factor, *c*, for an ideal refracting object. Then we examine the case observed experimentally.

For an LED that is placed far away from the sample plane, its illumination can be estimated as a spatially coherent plane wave:

$$U_{in}(\mathbf{r}) = Ae^{i\mathbf{k_n}\mathbf{r}}$$

where $\mathbf{r} = (x, y)$ denotes the spatial coordinates in the sample plane and $\mathbf{k_n} = \left(k_{xn}, k_{yn}\right)$ denotes the unique spatial frequencies based on each LED location. An LED array has a finite number of LEDs N_{LED} each with its unique location in the illumination plane (x_n'', y_n'') where $n = 1, 2, \dots N_{LED}$.

For a point where refraction occurs at an object (e.g. bead or cell edge), its interaction with the plane wave can be approximated as a thin lens with a variable focal length $f(\mathbf{r})$ introducing a quadratic phase shift given by the lens transfer function:

$$H_{object}(\mathbf{r}) = e^{-\left(\frac{i\pi}{\lambda f(\mathbf{r})}\mathbf{r}^2\right)}$$

Where λ is the wavelength. The focal length $f(\mathbf{r})$ is dependent on the object geometry and the refractive index at this location. The exit wave immediately after the object can be defined as:

$$U_{object}(\mathbf{r}) = U_{in}(\mathbf{r}) \cdot H_{object}(\mathbf{r})$$

Based on the convex shape of samples that we image; we assume the focal length is positive: $f(r) \geq 0$. Therefore, the object will introduce a negative phase shift towards the paraxial line located passing through the center of the lens. For two LEDs located exactly opposite of each other, the first is located at (x_0'', y_0'') and the second is located at the opposite location $(-x_0'', -y_0'')$. The object function will push the illumination coming from the first LED towards the direction of propagation and the other wave from the second LED away from the direction of propagation.

To propagate the exit wave to the pupil plane at a distance d from the sample plane, we utilize the Fresnel diffraction integral:

$$U_{pupil}(\mathbf{r}') = \left(\frac{e^{ikd}}{i\lambda d}\right) \int U_{object}(\mathbf{r}) e^{\frac{i\pi}{\lambda d}\left((r\prime - r)^2\right)} dr$$

Where:

- k is the wavenumber,
- $\mathbf{r}' = (x', y')$ denotes the coordinates in the pupil plane,
- The term $e^{\frac{i\pi}{\lambda d}\left((r\prime - r)^2\right)}$ is the Fresnel phase shift over distance d.

This wave is passes through the pupil function which acts as a low pass filter with a finite size (radius, $\mathcal{R}_{pupil}$):

$$P(\mathbf{r}') = \begin{cases} 1, |\mathbf{r}'| \leq \mathcal{R}_{pupil} \\ 0, otherwise \end{cases}$$

The filtered wave after the pupil function is:

$$U_{filtered}(\mathbf{r}') = U_{pupil}(\mathbf{r}') \cdot P(\mathbf{r}')$$

Then, the wave propagates through the system reaching the camera sensor. At the camera sensor (detector plane), the intensity is defined as the squared modulus of the propagated wave:

$$i_n(\mathbf{r}') = \left|\mathcal{F}\left\{U_{filtered}(\mathbf{r}')\right\}\right|^2$$

In darkfield imaging, the illumination source is positioned such that the incident wave at the pupil plane $U_{in}(\mathbf{r}_n')$ is outside the pupil function $|\mathbf{r}_\mathbf{n}'| > \mathcal{R}_{pupil}$. Continuing our two opposing LED example, if the phase shift by the object function results in the wave from the first being collected and producing an intensity, the wave from the second will not be collected and will not produce any signal.

Under a quadrant illumination (fig. 1c), a set of LEDs is used to illuminate the sample producing a partially coherent illumination mode, with each LED being mutually incoherent with each other. Therefore, the total intensity under a quadrant illumination is the linear sum of the intensities of single LEDs belonging to that quadrant:

$$I_c = \sum_{n \in Q_c} i_n$$

Where the cth image uses LEDs with indices Q_c chosen from $\{1,2, \ldots, N_{LED}\}$. $Q_1(BR)$ and $Q_3(TL)$ are composed of LEDs that are located exactly opposite to each other (fig. 1c). Based on the lens transfer function (negative phase shift) and the finite size of the pupil function, for a single point where Q_1 results in an intensity I_1, Q_3 should not result in an intensity I_3. For simplicity, we assume this point to be flat in the perpendicular plane i.e. no refraction which means I_2 (BL) and I_4 (TR) are equal to zero. By substituting back into equation 1, the edge signal (eqn. 1) becomes:

$$E = |TL - BR| + |BL - TR| = |I_3 - I_1| + |I_2 - I_4| = |-I_1| = I_1$$

For the darkfield image:

$$DF = TL + BR + BL + TR = I_3 + I_1 + I_2 + I_4 = I_1$$

Therefore, for a point with pure refraction, the QDF signal (the non-directional signal) is equal to zero. So, by substituting the previous two equations into equation 2:

$$QDF = c \times DF - E$$

$$QDF = 0$$

$$c \times DF - E = 0$$

$$c \times I_1 - I_1 = 0$$

$$\Rightarrow c = \frac{I_1}{I_1} = 1$$

However, experimentally, we find that the scaling factor, *c*, is not equal to one. For example, in images of beads, the opposing quadrant has a signal which can be observed by adjusting the color scale displayed.

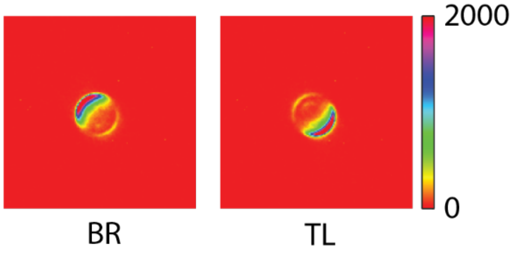

Figure S9. Quadrant images of clean polystyrene bead shown in Fig. 2. Colormap has been changed and the scale adjusted to show signal levels from the opposing edges of the bead.

When we quantify the signal at the opposing quadrant of the refracted signal in bead images (e.g. fig. S9), the signal in the opposing quadrant is approximately 10% of signal from the refracted first quadrant. Substituting back into the scaling factor equation:

$$c = \frac{|I_1 - I_3|}{I_1 + I_3} = \frac{|I_1 - 0.1\, I_1|}{I_1 + 0.1\, I_1} = 0.8$$

Thus, our scaling factor is a result of non-ideal light interactions that the refraction model does not account for. Such interactions include edge diffraction which occurs when light passes around the edge of a high refractive index material like a bead or a cell in our experiment. Edge diffraction (Rayleigh-Sommerfeld diffraction) results in the scattering of light close to the edge of the high refractive index material which can explain the weak signal at the edge of the bead. Other factors including surface roughness, especially in cells, sphericity of illumination wave, and imperfections in the system alignment are also not accounted for in our ideal system. A rigorous set of experiments and modeling is warranted to fully test and account for each of those effects on the QDF scaling factor.
